# AAPM working group on cybersecurity report 438: A white paper on cybersecurity management for business continuity in radiology and radiation therapy

**DOI:** 10.1002/acm2.70358

**Published:** 2025-11-23

**Authors:** R. Alfredo Siochi, Peter Balter, Samantha Hedrick, Jonathan Howe, Tianjun Ma, Emilie Soisson, Joshua Yung, Bruce Curran

**Affiliations:** ^1^ Department of Radiation Oncology West Virginia University Medicine Morgantown West Virginia USA; ^2^ Department of Radiation Oncology UT MD Anderson Cancer Center Houston Texas USA; ^3^ Medical Physics Thompson Proton Center Knoxville Tennessee USA; ^4^ Radiation Oncology Norton Cancer Institute Louisville Kentucky USA; ^5^ Radiation Oncology Virginia Commonwealth University Richmond Virginia USA; ^6^ Radiation Oncology University of Vermont Medical Center Burlington Vermont USA; ^7^ Imaging Physics UT MD Anderson Cancer Center Houston Texas USA; ^8^ Radiation Oncology VCU Health System Richmond Virginia USA

**Keywords:** business continuity planning, cybersecurity, disaster recovery, information technology, medical physics

## Abstract

A growing number of clinics have experienced disruptions of clinical services due to cyberattacks. To provide continuity of care, medical physicists should work with Information Technology (IT) staff to develop a business continuity plan. Such planning requires the participation of many stakeholders and should include the development of policies and procedures that not only make the clinic more cyber‐resilient but also address patient safety and ethics concerns. This report discusses the business continuity planning considerations for radiology and radiation oncology.

## 1 INTRODUCTION

### 1.1 Rationale and scope

Medical physicists play a key role in continuity of care (i.e., business continuity for healthcare), often in response to the effects of cybersecurity events, to enable clinics to continue to provide services despite the lack of appropriate data connectivity. Reports of such events at hospitals describe widespread outages.^1^ In one case, there was no access to the Electronic Medical Record (EMR) system for a month.^2^ In many cases, radiotherapy patients had to be treated elsewhere, causing delays in treatment. Such events can lead to prolonged overall treatment times, which negatively affect outcomes.^3^, ^4^, ^5^, ^6^ These events also decrease the availability of radiotherapy services within the local community. Similarly, the lack of access to a Picture Archiving and Communication System (PACS) prevents radiologists from providing timely input for the development of a patient's care management plan. While these events also have a monetary impact, such as the University of Vermont incident ($50 M),^2^ the focus here is on patient care.

Hospitals must have a system security plan, a comprehensive plan to address the security of all systems that exchange data. While the concepts in this paper are useful for the entire hospital, we limit the scope to those areas where the system's security plan is most likely to directly intersect with the medical physicist's clinical role. The plan should include the prevention and resolution of cybersecurity attacks and their effects. To do so, Information Technology (IT) staff will need to address the vulnerabilities in the existing IT infrastructure. While they can employ methods that make the system extremely secure, they should not compromise clinical functionality. This requires a dialog between IT, who understand the security issues and mitigations, and medical physicists, who have the technological expertise to describe the needed functionality and data transfers that are used to provide clinical services. Medical Physicists and IT staff need to develop a common understanding of the requirements and responsibilities for responding to a cyberattack. Additionally, for medical physicists to have meaningful discussions with IT staff, they must educate themselves on the basics of cybersecurity threats, modes of attack, preventative measures, effects on clinical operations, disaster recovery, and options for continuity of clinical operations. While some solutions are complex, some simple steps can be taken immediately to mitigate the effects of cyberattacks, as described by Yu, et al, and summarized towards the end of this paper.^7^

For these reasons, the AAPM (American Association of Physicists in Medicine) Working Group on Cybersecurity (WGCS) is addressing several important cybersecurity topics in this paper. While natural disasters, unintentional errors in IT operations (e.g., not excluding critical clinical systems from non‐tested operating system updates) and software bugs can cause clinical interruptions, they will not be discussed in this paper. However, applying the concepts in this paper will improve cyber‐resilience for these situations. This requires analyzing failure modes and their effects, and designing mitigations, like the work done by TG100^8^ (Application of risk analysis methods to radiation therapy quality management), but with a focus on the unavailability of data as the primary type of failure mode that interrupts clinical service.

The AAPM WGCS will focus on the description of cybersecurity events, and the professional aspects of business continuity planning as defined in this paper (see the definitions section and the section on cybersecurity threats). The overall goal of this paper is to encourage medical physicists to start conversations with their IT staff and their departments about business continuity planning and cybersecurity, and to provide them with the relevant information to do so. The recommendations in this report are not intended to be used as legal requirements. The details of the technical implementation of responding to radiation oncology related cybersecurity events will be addressed by the AAPM Task Group 393 (Radiation Oncology Contingency Plan Against Cyberattacks).

### 1.2 Definitions and concepts

Business Continuity: A state of continuous provision of services despite disruptions to normal operations. In healthcare, business continuity is the continuity of patient care.

Business Continuity Planning: The process of designing systems, policies, procedures, and activities that prevent disruptions, manage disruptions with contingency plans that define alternative processes that achieve the goals of the business, restore normal operations and systems, and train staff to implement the contingency plans. The four components of a Business Continuity Plan (BCP) are Prevention, Preparation, Response, and Recovery. Prevention involves identifying the essential activities and resources of the clinic, understanding the clinical workflow as it exists (not as it was designed), identifying threats, and mitigating them to reduce the probability that the threat will occur. Preparation involves designing alternative processes, testing them, and rehearsing them with a cyberattack drill so that in the event of a disaster, all involved personnel know their roles and tasks. In the Response phase, key personnel manage the crisis, assess the damage (such as what data was lost), and use a communications plan to outline what alternative processes, data centers and networks will be used, triage patients, manage the damage and its impact on each patient, and describe the timeline of response and recovery operations. In the Recovery phase, IT infrastructure and clinical processes are restored to a state equivalent to or better than the state prior to the disaster. In this phase, lost data is either recovered or reconstructed, and network communications are restored.

Cybersecurity: protection of networks, devices, and data to ensure its availability, integrity, authentication, and confidentiality. (Paraphrased from the NIST glossary.^9^)

Cyber Device: a device that can connect to the internet and has characteristics that could be vulnerable to cybersecurity threats. The legal definition in the FD&C act^10^ is more nuanced, but this definition suffices for the discussion in this paper.

Disaster Recovery: This term is often used to refer to Business Continuity when the interruptions are due to a disaster such as a fire or a flood. It is also sometimes used to refer to the Response and Recovery components of a BCP. In the context of this report, the disaster is a cyberattack.

File Mode: A mode in which an offline treatment (see Offline Procedures) can be performed on a True Beam linear accelerator (Varian Medical Systems, Palo Alto, CA) by loading a locally stored DICOM‐RT file with the treatment plan information.

Offline Procedures: The performance of procedures without being connected to the hospital network. In radiation therapy, these are often referred to as offline treatments. Linear accelerators may have several components that are networked together in a local area network (LAN), but their LAN is isolated from the rest of the hospital network, typically by a firewall, that limits communications to the minimum required for the linac to operate. When the hospital network is down, the linear accelerator can still deliver radiation—this is sometimes called offline mode or standalone mode. In a radiology department, scans can still be performed on imaging equipment, but they cannot be transferred to the hospital Picture Archiving and Communications System (PACS). Radiologists may have to view the images at the scanner rather than at their normal workstations.

Service Mode: A mode that is intended for service engineers and is not intended for treatment. It is possible to perform an offline treatment in service mode by manually entering machine parameters, but several safety checks may be lost compared to a treatment in clinical mode.

Standalone / offline Mode: See Offline Procedures.

### 1.3 Cybersecurity threats

According to the National Institute of Standards and Technology (NIST), cybersecurity threats are any circumstances or events with the potential to harm an information system through unauthorized access, destruction, disclosure, modification of data, and denial of services.^11^ Specifically for the healthcare industry, the consequence of a successful cyber‐attack may result in both data breaches and disruption of services, such as loss or disclosure of patient‐related information, disruption of patient care or even potential malfunctioning of medical devices. These threats can be internal or external.^12^

Internal cybersecurity threats originate within an organization itself, such as from current or former employees, contractors, or vendors. The insiders who have access to organization data/devices could impact the information system with malicious intent or normal actions that have unintended consequences. Examples can vary from sharing unencrypted data with unauthorized parties, unauthorized access of sensitive data, falling victim to social engineering, and physical loss or theft of an unencrypted organizational device.^13^

External threats originate outside of an organization, such as from hackers and cybercriminals. They can exploit system vulnerabilities (both software and hardware defects) through various attack vectors such as phishing, malicious codes, and denial of service (DoS), resulting in profound consequences for data security, business continuity, organization reputation, patient treatments, and customer satisfaction.

Cybersecurity threats can arise not only from human actions, but also from events that are outside of the control of an organization (e.g., natural disasters, unavailability of critical infrastructure).^14^ These threats are constantly evolving. To be aware of current cybersecurity threats, cybersecurity threat intelligence (CTI) can be applied by an organization for better monitoring and preparation.^15^ Improved monitoring is crucial, as the number of incidents continues to increase. (The rate of ransomware attacks in 2023 was about twice what it was in 2021.)

## 2 APPROACH

Part of our responsibility as medical physicists is to maintain clinic operations, from a technical perspective, which requires us to understand and implement the key concepts associated with Business Continuity Planning. We must identify cybersecurity threats that could disrupt the delivery of services and design our IT infrastructure and clinical operations to (1) prevent attacks from reaching the network, (2) where possible, minimize the effects of such attacks, (3) continue performing procedures possibly using backup systems in the event an attack disables the primary clinical system, and (4) restore the primary clinical system and backup system to their required states. These activities must be done in a time that limits the effects of interruptions to a level that is acceptable.

To achieve these goals, we need to learn from those who have experienced cyberattacks. The WGCS met once a month and discussed the events that some of the members experienced firsthand. We studied related events that have been published in radiation oncology and radiology related journals.^2^, ^7^, ^16^, ^17^, ^18^, ^19^ Four of these events are discussed in Section 3, “Examples of Cybersecurity Events”. Several of our members have developed, or are developing, business continuity plans. The goal of these plans is to become cyber‐resilient, as discussed in Section 4. While discussing recommendations, controversial suggestions were reviewed from an ethical framework, as described in Section 5.

**FIGURE 1:**
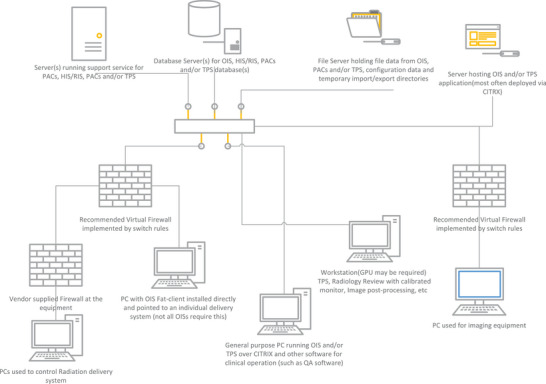
A generic networking diagram showing equipment, servers, and firewalls employed in clinical networks supporting Diagnostic Imaging and Radiation Oncology.

While the existing literature has published recommendations, this paper focuses on the professional aspects of Business Continuity and encourages medical physicists to lead their departmental efforts in BCP. The first step in this process is to understand how data flows within the department. A generalized departmental data flow diagram is presented in Figure 1 and in Section 6, where IT equipment and networks for radiation therapy and radiology departments are described in terms of the data transfers and computing operations that support the essential clinical operations. Understanding these data flows also helps to identify the consumers of this data, who are described in Section 7, “Stakeholders.” Section 8 provides recommendations on the roles and responsibilities for various BCP tasks in tabular format. Several of these responsibilities belong to the hospital IT department, and have been listed in Appendix A to serve as a minimum checklist for medical physicists to discuss with IT; every hospital IT department will have its own cybersecurity policies and procedures, but the checklist was developed to ensure that clinical functionality is considered by IT. Finally, Appendix B provides a general framework and a use case example of its application to one of the cyber incidents in Section 3.

## 3 EXAMPLES OF CYBERSECURITY EVENTS

We can learn from recent cybersecurity events, such as the hospital network wide outage at the University of Vermont Medical Center that affected six hospitals in Vermont and New York. In another incident, a major player in radiation therapy solutions suffered several cyberattacks that affected over 40 healthcare systems that accessed their Cloud Data Storage. One of the healthcare systems, Yale New Haven Health, was unable to treat over 200 cancer patients for more than a week and had to transfer patients to other hospitals in the area.^17^ At the Provision Center for Proton Therapy, a ransomware attack disabled desktop computers, Oncology Information Systems (OIS), Treatment Planning Systems (TPS), phones, and the shared network on a Monday, but they were able to treat 40 proton patients by Friday. The description of the events at the Provision Center comes from one of the authors who experienced the cyberattack firsthand. In 2020, a ransomware attack affected one of the hospitals in the tertiary referral network of the Cleveland Clinic Imaging Institute, and they were disconnected from the network, requiring the use of paper‐based workflows for diagnostic radiology operations.^19^

### 3.1 University of Vermont

The details of the attack at the University of Vermont Medical Center (UVM) in October 2020 were described by several news outlets, and its impact in radiation oncology is very well documented by Nelson, et al.^2^ While on vacation, an employee using a company laptop accessed a personal email from a legitimate business that was hacked in a broad phishing attack. When the employee connected to the hospital network via VPN (Virtual Private Network), an email attachment infected with malware was used to launch the attack. The IT service desk at UVM started getting numerous calls about software glitches and quickly realized they were the result of a cyberattack. Hospital IT responded with an immediate shutdown of the internet and EMR for the entire network, removing access to phones, email, PACS, pathology, pharmacy, physics databases, QA (Quality Assurance) software, and all patient contact information. As patients arrived for their cancelled treatments, staff requested their contact information. Fortunately, the treatment planning system was Unix‐based and was not affected by the attack. CT (Computed Tomography) images could still be acquired, and the DICOM files were rerouted to communicate directly with the planning system. The process of bringing computers and servers back online was slow and had to be done with an ongoing cyber‐forensics investigation. The radiation oncology department was told to prepare for an extended loss of the OIS, requiring immediate triaging of patients who were either sent to another network site or delayed, depending on urgency.^16^ Some simple cases were treated with offline operation of the linac. The safety concerns of this approach were considered against the risk of poor patient outcomes resulting from prolonged treatment interruptions. To mitigate the safety concerns, a system of checklists and safety rules were developed, such as requiring a physics presence at the machine to ensure treatment accuracy.^2^ Since it was not possible to transfer all patients, UVM had to set up a standalone Radiation Oncology Information System (ROIS) server, export the treatment plans from the unaffected planning system and re‐import all planning information into the standalone ROIS, aka the “downtime OIS”. Prior treatment information was not known, but could be determined from patients via conversations and their printed calendars. Within 2 weeks, patients were treated in the “downtime OIS”. Treatment continued in the downtime system until January, when the legacy database was finally restored. It is estimated that the attack cost $40–50M. The EMR was restored about a month after the attack, and radiology viewing was restored 40 days after the attack.

### 3.2 Yale new haven health

At Yale New Haven Health (YNHH), patient data for linear accelerators were stored in a cloud platform. To treat a patient, the staff accessed the cloud to download the treatment plans to the linear accelerator. When the cloud platform experienced a breach, due to unauthorized access by a third party that used compromised credentials,^20^ access to the cloud was removed to prevent the malware from spreading to their customers. As a result, YNHH lost access to patient radiotherapy plans and could not deliver treatments. It took over a week to move the data from the affected first‐generation cloud system to their new cloud and restore YNHH's access to their patient data. During that week, YNHH triaged patients; patients who were at greater risk of poorer outcomes due to protracted interruptions were treated either in File Mode or transferred to a nearby unaffiliated hospital with a beam matched linac. For the File Mode capable linacs in the system, procedures, like those employed by UVM, were developed to treat in File Mode. In the meanwhile, YNHH decided to build a local ROIS server from scratch, with linacs treating on the new server within 13 – 20 days.^17^

### 3.3 Provision center for proton therapy

At the Provision Center for Proton Therapy (PCPT), six proton treatment rooms, at two locations, and a single linac were affected by a ransomware attack. Systems began to go down on a Monday and, by Tuesday were entirely down. The linac was unable to treat in stand‐alone mode. The proton treatment delivery system was unaffected, protected by firewalls and the use of Linux.

It was discovered that the proton system could be treated in a stand‐alone mode, with plan data for patients under treatment saved on the treatment delivery system. The proton center was treating 40 patients per day by that Friday. Employee's personal webcams were used, with cables running under the treatment doors, to visualize patients during treatment. Paper charts were created using plan data collected for scripting purposes on a separate network. The TPS vendor sent a stand‐alone server and license, allowing the center to continue treatment planning. CT data was transferred via USB drive to the TPS, and plan data for new patients were transferred to the proton delivery system also via USB drive. The USB drives were checked for malware before each use. Patients were consented to treat outside the normal use of safety interlocks that did not exist in stand‐alone mode. A backup of the OIS and TPS were eventually recovered and restored.

### 3.4 Cleveland clinic imaging institute

A hospital within the Cleveland Clinic's tertiary referral network experienced a ransomware attack in 2020. To prevent the ransomware from spreading to the rest of the hospitals on the Cleveland Clinic's enterprise network, the affected hospital was disconnected from the network. As a result, they were unable to use their normal workflows that relied on communications across the network; to continue their operations, radiologists wrote orders and reports on paper and stored diagnostic images on CDs and DVDs.^19^ They viewed images on the console of the imaging device. They communicated by phone and fax. Once the network became available, for each patient, the images and documentation created during the downtime had to be reconciled with the rest of their chart. Images had to be manually sent to the PACS, and reports “re‐dictated” so they appeared in the EMR.

### 3.5 Lessons learned

While the incidents previously described can be avoided by following improved IT security protocols, ransomware attackers are continually finding new ways to break through a facility's security mechanisms. Avoiding the attacks in the first place requires a thorough vetting of the vendor's systems that are involved in data transfers, with particular attention to cloud platforms.

If an attack should affect radiation oncology, staff should be prepared to continue treating patients; backups of patient contact information, schedules, and treatment plans are essential. Health systems should use standardized treatment and planning software and devices to facilitate the transfer of patients amongst the various system facilities, while other radiotherapy clinics should consider partnering with nearby clinics with similar equipment. Proactive discussions with vendors are needed to ensure that well‐defined procedures and systems are in place to serve as an alternative means for treating patients. Such systems need to consider all aspects of patient care, including the ability to monitor patients during treatment and to verify the correctness of the delivered treatment as planned. Safe ways must be found to operate treatment devices in standalone mode, or to overcome those limitations that prevent offline treatments. As these ways can be cumbersome and time consuming, focusing on patients whose outcomes will suffer the most because of the treatment interruption is imperative. This triage and prioritization strategy must be part of the Business Continuity Plan. This plan must also describe how to recover from the event and provide methods for reconstructing the downtime treatments in the patient's chart when data connectivity is restored. TG‐314 on Fault Recovery provides recommendations for resuming treatments safely after an unplanned downtime, many of which are applicable to recovering from a cyberattack.^21^

Radiology departments must have alternative workflows to order imaging examinations, prepare and send reports, store images, provide images to other physicians, and communicate within the department, with patients, and other providers in the event of a cyberattack. Remote reads of images are not possible, and alternatives to remote locums’ coverage must be found since images must be read at the scanner. The Cleveland Clinic Imaging Institute and Information Technology Division developed a comprehensive set of readiness checklists for radiologists in each subspecialty, created ordering and reporting templates, developed an extended downtime manual, and wrote a continuity of operations playbook. They organized their continuity planning considerations according to four phases: (1) Hyperacute (first 48 h), (2) Acute (first 3 weeks), (3) Infrastructure Recovery, and (4) Reconciliation. For the full details of their “Lessons Learned and Planning Considerations,” see the work of Chen, et al.^19^

With the loss of data connectivity, remote work arrangements may not be possible. For staff who live several hours away, it may not be practical to participate in the immediate response to the cyberattacks. Departments should develop alternative staffing models and remote work responsibilities so that the remote FTEs can work on tasks not requiring the network, (or use alternative data connections), and the on‐site tasks can be performed. An example in radiation therapy would be remote treatment planning by dosimetrists; an example in radiology would be remote reads of exams.

## 4 CYBER‐RESILIENCE

The events in the previous examples could have been avoided if the department's systems were cyber‐resilient. These systems must be able to recover quickly from all types of cybersecurity threats, whether internal with unintended consequences (such as the release of system patches that are incompatible with clinical devices) or external threats such as cyberattacks. Establishing a strategy for cybersecurity preparedness can be overwhelming given the complexities of healthcare technology, infrastructure, and workflows.

There are tools to help. NIST has developed a cybersecurity framework^22^ that can be applied to a broad range of healthcare systems, and business units. This framework includes the following components: Identify, Protect, Detect, Respond, and Recover. When utilizing this tool, it is recommended to develop a base strategy for each component and periodically revisit it to build on the foundation. In this way, the preparedness plan will continue to be updated, expanded, and improved for robustness while ensuring recent technology is included.

The FDA and IEC also provide cybersecurity resources for manufacturers. The FDA offers guidance on managing security risks to medical device manufacturers in a draft guidance document.^23^ The IEC provides standards for the security of industrial automation and control systems (IEC 62443^24^), information security management systems (IEC 27001^25^), and cybersecurity (IEC 27032^26^). While these documents are aimed at manufacturers, consumers should consider upgrades and purchases that take advantage of the improved security that results from manufacturers implementing these standards.

Reviewing processes for system recovery, roles, responsibilities and expected time to recover should be discussed as part of department routine meetings. Resources to aid in discussion include checklists,^18^ tabletop exercises^7^ and the information and prepared learning tools from the Department of Health and Human Services,^27^ which also provides scalable resources for small to large healthcare organizations.

## 5 ETHICS

Even the most cyber‐resilient institutions must face the possibility of a cyberattack that disrupts the usual workflows for patient care and results in resource shortages. It may be necessary to operate under a “crisis standard of care” as defined by the National Academy of Medicine (NAM) in 2012.^28^ This refers to an “optimal level of care that can be delivered during a catastrophic event, requiring substantial change in usual health care operations.” Many federal, state, local agencies, and other entities (including the NAM) have recently revised crisis standard of care guidelines because of the COVID‐19 pandemic.^29^ During the pandemic, there were shortages of ventilators and other medical resources, and it was important to establish an ethical framework to deliver the best care for the greatest number of people. These guidelines provide a framework for medical practitioners to manage scarce medical resources in a crisis.^30^

In normal times, it is best to treat patients with the current standard of care. When something unexpected happens, though, it may be necessary to move to contingent care with the expectation of the same outcome for all patients, with a slight change to procedures due to some external factor. One may be forced to apply a workaround or non‐standard procedure to achieve the same result with the goal of ensuring the care is equivalent despite the modification.^31^, ^32^ In a crisis, care needs to be significantly altered, and a risk of adverse outcomes for patients is introduced. In this case, the focus moves from providing the best care to each individual patient to getting the best outcome for the entire population of patients or providing the best care to the greatest number of people.

While the idea of triaging care is common in emergency rooms and emergency response, it is less common in radiation oncology departments. Therefore, in a crisis, providers may not be familiar with an ethical framework to guide treatment decisions.^33^ In medicine, NAM provides educational materials for medical providers to handle the delivery of care in a resource limited setting based on the following core values:^34^

‐ “Fairness: treating each patient with equity and evaluating them in the same way
‐ Duty to Care: for each patient, without bias, to the best of your ability
‐ Duty to steward resources: to do the greatest good for the greatest number
‐ Transparency: to have shared assumptions, processes, and documentation
‐ Consistency: to provide a consistent level of care within a facility and region
‐ Proportionality: to only restrict care to the degree that we must, no more
‐ Accountability: to engage experts as needed and document our decisions and our process”

Physicians and medical physicists should familiarize themselves with these tenets ahead of a crisis so a method of triaging patient care can be quickly established in the event of a cyberattack. These core values should also guide the department's BCP.

While altering care may have legal ramifications, there are certain legal protections offered at the federal and state level to protect health care workers in the case of a good faith implementation of a crisis standard of care.^35^ In general, withholding care due to the fear of legal ramifications could result in worse outcomes for patients than delivering modified care, so it is important for the treatment team to do the best they can with the resources available.

Since the risk of cyberattacks and their impact on oncology operations is well known, radiation oncology departments have an ethical responsibility to prepare for this situation. As part of this, an understanding of local and federal laws and how to fairly triage patients and manage risk should be obtained prior to an attack.

Here are some ethical issues and responses to consider:

- Should patients be notified of the attack?
  ○ Patients will eventually hear of the situation in the news. It is best to let them know that their care is being managed despite the cybersecurity issues.
- Should we treat patients?
  ○ Sending patients elsewhere may require new insurance authorizations, simulations, and treatment plans, requiring several weeks of turnaround time. One should avoid delays of care that could compromise their outcome. If a safe treatment workflow alternative can be implemented quickly, such as a standalone mode treatment, it would be better to treat them than to send them elsewhere.
  ○ For patients not yet simulated, give them the option of going somewhere else or waiting a little longer to figure out the process (a couple of days). High‐risk patients who cannot afford any delays may need to be simulated and treated elsewhere.
- Should patients be notified that they are being treated without normal interlocks?
  ○ Yes. This can be done through an addendum to the consent, explaining the risk of treating in standalone mode versus the risk of not receiving timely treatment. The explanation should also mention that alternative procedures have been developed to perform mitigations equivalent to those of the interlocks. This is consistent with the core value “Duty to Care.”^34^
- Should the state be notified of the situation?
  ○ Consult with your institution's legal department. In one scenario, a clinic that was treating outside of their approved standards sent a letter to the state, notifying them of treating in standalone mode.
- What if your institution had a HIPAA (Health Insurance Portability and Accountability) guideline that forbids the opening of shred bins, but you had no other access to information to contact patients to alert them to the situation?
  ○ HIPAA does not prohibit access to patient information if it is necessary for a health care provider to do their job. Guidelines that interpret HIPAA for specific situations need to be reviewed for exemptions during cybersecurity events. During a cyberattack, individuals needing access to information should seek some form of expedited exemption to the policy while still respecting HIPAA regulations.

It is common to struggle with these decisions, but applying the core values of the crisis standards of care allows one to act in good faith and in the best interest of all patients.

## 6 DEPARTMENTS

Figure 1 shows a generic network diagram for Radiation Oncology and Radiology. Servers are shown at the top of the figure. They span one or more data centers with double or triple redundancy (not shown for clarity). The support servers on the top left run services related to application operations and maintenance such as importing and exporting data from the database(s), HL7 processing, webservers for application management, etc. The database servers can be distinct, application specific servers or can be an institutional database cluster that hosts the databases used by ROIS, PACS, QA systems, etc. The file servers hold data needed to run the applications, often as a logical segmentation of a network‐attached‐storage (NAS) or storage‐area‐network (SAN), which includes configuration information, in some cases the executable for the applications, buffer space for processing, large objects not saved in the database as well as input and export buffers. The servers on the right are application servers used to remotely run clinical software via CITRIX, web apps, or other virtualization systems. These systems are connected via the facility WAN which may span multiple buildings or multiple clinics across a region. Medical equipment can be directly on the WAN, but the preferred implementation would use a firewall between the medical equipment and general network traffic. Several pieces of related medical equipment may share a common firewall and can communicate with each other with no restrictions. Some vendors include hardware firewalls into their equipment to separate internal network communication from the facility network which adds a level of security, allows simplification of equipment design without customization for each customer network, and can allow the vendor to reduce the amount of operating system (OS) patching needed to keep systems safe due to a very restricted amount of network traffic.

## 7 STAKE HOLDERS

An organization's management of cybersecurity threats requires identifying stakeholders and defining their responsibilities. The stakeholders include healthcare IT support staff, medical physicists, nurses, radiation therapists, imaging technologists, engineers, physicians, administrative staff, including clerical and billing staff, and the vendors supplying equipment and/or software. The design of a cyber‐resilient system should consider the needs of each of these groups, otherwise workarounds will be developed that will be detrimental to the overall effort. Table 1 provides a description of the stakeholders.

**TABLE 1:** Descriptions of stakeholders.

Stakeholder	Description
Healthcare Information Technology (HIT)	HIT manages user authorization, networks, storage, servers, databases, and applications support. Different subgroups within HIT may be assigned to manage one or more of these functions. HIT may manage other functions, but the items listed here are managed in partnership with medical physicists.
Medical Physicists (MPs)	MPs ensure the accuracy of data for diagnosis, planning, and treatment. They verify that data is correctly exchanged among several medical devices and systems. They are critical in reviewing system inter‐dependencies, determining what services can be delivered if one or more systems are off‐line, and developing safe clinical workflows for those services.
Physicians (MDs)	MDs and their support teams (APPs, PAs) use systems for diagnosis or treatment.
Nurses	Nurses assist MDs in the preparation for patient consults and exams and provide supportive care to patients during their course of treatment. They need to be trained to use paper‐based workflows and/or alternative workstations in the event of a cyberattack.
Radiologic Technologists (RTs)	RTs set up and image patients. They need to be trained to use paper‐based workflows in the event of a cyberattack.
Radiation Therapy Technologists (RTTs)	RTTs set up and treat patients. They need to be trained to treat using alternative methods in the event of a cyberattack.
Certified Medical Dosimetrists (CMDs)	CMDs create radiotherapy treatment plans. They may need to use alternative workstations and alternative data transfer methods in the event of a cyberattack.
Non‐vendor engineering support	This includes IT staff and biomedical engineers of the department (not the hospital), and third‐party groups that provide service and maintenance to the systems covered in this document. They are vital not only for ensuring systems are kept up to date (e.g., security patches), but also for recovery from a cyber disruption.
Administrative staff	This includes receptionists, schedulers, billing personnel, and their supervisors. They need to be involved in the development of alternative workflows to register, schedule, identify, check‐in, and bill patients.
Administrative leadership	This includes those responsible for budgeting, staffing, organizing and strategic planning. They should ensure resources are available to provide business continuity in the event of a cyberattack.
Vendors	Vendors provide hardware and software for systems described in this document. The primary vendors provide medical equipment and software such as imaging, treatment, and planning systems. These vendors often rely on secondary vendors who provide computers, operating systems, drivers, GPUs, remote access/monitoring software, data storage, and the like. It is the primary vendors' responsibility to understand vulnerabilities from the secondary vendors and inform the customers and help implement mitigation strategies.

## 8 RESPONSIBILITIES

Departments should form a working group for BCP (WGBCP) composed of at least one representative from each of the stakeholders in Table 1. This working group should address the four phases of BCP (Prevention, Preparation, Response, and Recovery). They should design the alternative workflows that are needed to maintain critical operations in the Response phase and define the roles and responsibilities of each stakeholder in each phase. They should keep in mind the concepts of Ethics and Cyber‐resilience (Sections 4 and 5) when they design the BCP. They should also consider the patient's privacy and confidentiality in the BCP, as alternative workflows may be needed, and third parties may have to access clinical systems for service. The department and the vendors should make sure they have the appropriate Business Associate Agreements in place.

The WGBCP can use the tables in this section as a starting point for their design of the BCP. These tables define a minimum set of responsibilities for each of the stakeholders within the department. Although HIT responsibilities are determined by the hospital's IT department and leadership, the WGBCP needs to work with them to ensure that details specific to the departments’ operations are considered by HIT. A table of HIT responsibilities is provided in Appendix A. It can be used as a checklist and talking points for discussions with HIT.

Table 2 Lists the responsibilities of the Medical Physicist, as it pertains to cybersecurity issues. Responsibilities are listed by category and BCP phase. For other responsibilities, see AAPM MPPG 10.a for the scope of practice for clinical medical physics.^36^ Tables 3, 4, 5 provide cybersecurity related business continuity responsibilities of Physicians and their support staff, the Administrative Staff and Administrative Leadership, respectively.

**TABLE 2:** Cybersecurity responsibilities of the medical physicist.

Category / Phase	Responsibility
Technology selection / Prevention	Work with HIT and vendors to deploy robust solutions that both meet the patient's needs and limit the vulnerabilities to cyber disruption.
Informatics / Prevention	Medical Physicists or Software Developers working with Medical Physicists should verify that in‐house code is free of bugs that may disrupt clinical operations or introduce vulnerabilities to cyberattacks.
	Work regularly with HIT and vendors to ensure systems are being maintained and patched and that this work does not interfere with the clinical operations of the systems.
Informatics / Preparation	Know the system's architecture of the radiation oncology and radiology infrastructure, the connectivity between the different components, their purpose, and where data is stored. Create a diagram like Figure 1 with all the actual devices in the department.
QA / Response and recovery	Verify that data integrity is maintained, calculation algorithms are verified, and data flows as expected^37^ with an end‐to‐end test after each change that could affect them, such as a switch to failover systems in the response phase or replacement systems in the recovery phase.
Administrative / Preparation	Work with HIT to develop a Departmental downtime manual with contact lists, emergency call tree, network and data flow diagrams, and alternative workflows.
Administrative / Response and recovery	Bridge the needs of HIT and the physicians in planning for a response to cyber disruptions and develop a recovery strategy after an attack.
Clinical / preparation	Work with HIT and physicians to develop and test off‐line workflows.
	Work with HIT to set up cyber‐attack drills. This should test all elements of the BCP. Coordinate with the WGBCP to prepare staff for the drill.
Clinical / Response	Provide on‐site support for all technical staff during an event, as they may be using unfamiliar systems, workflows, and/or dataflows
	Ensure any treatments occurring during down‐time procedures are properly recorded
Clinical / Prevention, preparation, response	Work with HIT to ensure that removable media used for file transfers is not a vector for malware; use security rules on restricting executing code from the media.
Clinical / Recovery	Reconcile treatments and/or procedures for each patient so that the total dose during downtime is accounted for in the ROIS and/or the EMR.
	Reconstruct the patient's chart in the ROIS and/or the EMR to ensure that it reflects all care provided before, during, and after the cyberattack.

**TABLE 3:** Cybersecurity responsibilities of physicians and their support staff.

Category / Phase	Responsibility
Administrative / Preparation and response	Radiologists should ensure systems are in place to provide critical imaging and interventional services that cannot be delayed until the cyber disruption is over. Radiation oncologists should ensure systems are in place not only for the intake of new critical patients but also continuity of care for patients under treatment.
Clinical / Preparation	Form partnerships with other hospitals and clinics that could image or treat their patients in the event of a cyberattack.
	Develop a triaging system that can be used immediately in the event of a cyber disruption. This could be done by documenting a triage group in an Orders document (e.g., priorities are 1 = high, 2 = medium, 3 = low) for each patient. This process should be integrated into the clinical workflow for normal operations.
Clinical / Response	Review the patients under care to create triage lists from the triage groups in the patient's documents.
	Plan for patients to be imaged or treated at another hospital if there is potential for an extended downtime.

**TABLE 4:** Cybersecurity responsibilities of administrative staff.

Category / Phase	Responsibility
Informatics / Preparation	Work with HIT to identify all the information systems they use for registration, check‐in, scheduling, billing, calling patients and any other tasks assigned to administrative staff.
	Work with HIT to develop alternative workflows and systems. HIT may have solutions for standalone EMR access with day old data. Other systems may require paper‐based solutions.
Administrative / Preparation	Maintain up‐to‐date contact lists for staff and for patients with appointments for the following week.
	Manage communications with patients during the downtime.

**TABLE 5:** Cybersecurity responsibilities of administrative leadership.

Category / Phase	Responsibility
Administrative / Prevention	Review BCP plans to prioritize robustness of operations.
	Work with HIT to budget for equipment upgrades and replacements that will reduce vulnerabilities.
	Ensure all groups have the time and resources to develop and deploy a business continuity plan.
	Work with HIT to develop a budget for failover systems, backups, and other solutions needed to maintain operations during a cyber related disruption.
	Manage the cyber‐resilience education and training of all staff.
Administrative / Response	Work with the WGBCP to inform the department of the cyberattack, and to discuss the logistics of the response with staff.

Tables 6A, 6B, and C list the responsibilities of vendors of cyber devices. Table 6A applies to all systems, while Tables 6B and C cover portals, clouds, and offline treatments. MPs and HIT should consider these lists as a set of guidelines for evaluating the cyber‐resiliency of software and hardware products. Vendors should use these lists to design cyber‐resilient solutions, and to upgrade existing systems to make them more cyber‐resilient. The WGCS encourages vendors to adopt these recommendations. Note that some of these recommendations are required by section 524B of the FD&C Act 10, or are recommended by the FDA in a guidance document^38^ that addresses compliance with section 524B. A number of these recommendations protect patients from cyberattacks. FDA related recommendations are marked with an asterisk in the responsibility column.

**TABLE 6A:** Cybersecurity responsibilities of vendors—All systems.

Category / Phase	Responsibility
Design / Prevention	*Design systems to limit vulnerabilities.
	Consider including a hardware and/or software security system to limit the possibility of loss of function or data, especially when the operating system is not being regularly patched.
Design / Response	Provide the ability to treat using locally cached data, newly created DICOM files and/or manual machine settings. This could be a preconfigured local server to provide temporary support during the outage or could be an offline treatment mode.
Maintenance / Prevention	*Support products over the expected useful life, with upgrades and patches to address new cybersecurity vulnerabilities as new threats become known.
	*Immediately report any new threats to all customers, and the steps being taken to address them, even if there is no supplied mitigation available yet.
	*Monitor and manage the vulnerabilities that could be introduced by systems from third parties.
	Avoid restrictive Food and Drug Administration (FDA) clearances (510K) that limit the ability to patch and update where possible.
	*Provide a means of virus protection or give guidance to the customer on how to provide this without affecting the functionality and performance of the system.
	*Proactively provide the “Manufacturer Disclosure Statement for Medical Device Security”^39^ (MDS2) forms to the customer, update these as needed, and push the updates to all known customers.
	*Provide a software bill of materials (SBOM) so that third party vulnerabilities can be tracked.
	*Provide guidance and/or support for removing all Protected Health Information (PHI) off a system when parts are replaced, or a system is decommissioned including returning demo systems or loaned equipment.

**TABLE 6B:** Cybersecurity responsibilities of vendors—Portals and cloud solutions.

Category / Phase	Responsibility
Design / Prevention	Isolate patient portals from their associated oncology information system (OIS) or hospital information system (HIS) to prevent the introduction of new threats into the OIS or HIS.
	Manage the upload of patient objects to the patient portal to ensure that they are not contaminated with malware.
	*Provide patient portal authentication systems, including the use of dual factor authentication, to prevent bad actors from gaining access to the portal.
Design / Preparation	Vendors with cloud options for their OIS are encouraged to have not only a local fallback mode in case the cloud becomes unavailable but also an option for a cloud fail‐over mode in case a locally hosted system becomes unavailable.
Design / Response and Recovery	Provide an option for a pre‐configured cloud instance that could be rapidly deployed in case a locally hosted OIS becomes unavailable. Local and cloud databases should be synchronized within minutes. An alternative is to have a blank cloud database with machine and clinic information but no patient data, and a means of combining treatment records in this database back to the clinic main database once normal service is restored.

**TABLE 6C:** Cybersecurity responsibilities of vendors for offline treatment support.

Category	Responsibility
Design / Response	Systems that can operate in DICOM/file mode should have provisions for use with limited or no access to external identity management systems and/or network (e.g., have a local “emergency account” that can be used for treatment in these circumstances, or locally cached credentials)
	Support full stand‐alone operation of their treatment units based on manual treatment and/or locally cached DICOM files.
	Provide a means of allowing access to DICOM files via a network share that can be adjusted without vendor intervention (not requiring restricted controls to update the share location) or some type of removable media.
Design / Response and recovery	Provide a means for treatments and images to be locally stored during a cyberattack and uploaded into an OIS when normal operations are restored.
Support / Response	Vendors should work closely with clinical staff to support business continuity efforts during a cyberattack. An example of this type of support was demonstrated by one TPS vendor who sent a laptop and a license for treatment planning, enabling the dosimetrists to continue planning.
	Vendors should work closely with clinical staff to find out what processes have been interrupted and discuss ways of resuming them. Part of this discussion should include customer questions about affected hardware and software.

Vendors strive to support better patient experience through patient portals, better integration of equipment through expanded interfaces with the OIS, and more versatility with offering both cloud and on‐premises hosting of systems. All these methods to improve patient experience have implications for cyber resilience. Table 6B lists recommendations for cyber‐security for patient portals and cloud solutions.

Vendors have also increasingly been requiring real‐time access to an ROIS to perform even basic treatments and/or authentication for any equipment use. This can create issues for performing procedures if access to the ROIS is lost. Service mode treatments can be used with appropriate oversight and proper review of the relative risks versus not treating, but it is not advisable as it forces the team to work in an unfamiliar user interface (UI) with many of the safety features bypassed. If there are no other options, additional clinical processes should be implemented to mitigate the loss of the safety features. To avoid this situation, the vendor should follow the recommendations in Table 6C, cybersecurity responsibilities of vendors for offline treatment support.

Vendors should also have a means of providing backwards compatibilities during upgrades, especially in health systems whose clinics may have different versions of systems and may not be able to upgrade. While not related to cybersecurity, this speaks to business continuity for those systems that cannot be upgraded.

## 9 CONCLUSION

While it is best to prevent a cyberattack, one must be prepared to address interruptions caused by such attacks. This document provides an overview of the ethical framework needed to design a BCP. Departments should make a list of the stakeholders and their responsibilities in such plans, along with diagrams and tables of information showing the normal data flow through various systems, and the altered data flow resulting from the implementation of the BCP. The BCP should also consider the lessons learned from other institutions that have experienced cyberattacks, such as the importance of a communication plan, patient triaging, alternative clinical workflows, the use of backup systems, and proactive discussions with vendors to develop methods for using their systems when the normal network connections are not available. The Working Group on Cybersecurity hopes that this report will facilitate the discussions at institutions on the development of Business Continuity Plans, and that medical physicists will educate hospital management and IT professionals on the unique needs of our data‐intensive practices.

## AUTHOR CONTRIBUTION

Alf Siochi wrote the introduction, conclusion, part of the examples, assembled and edited the manuscript and managed the writing of the paper. Peter Balter wrote the section on stakeholder roles and responsibilities, and the section on network configurations. Sammie Hedrick wrote part of the example section. Jonathan Howe wrote the cyber‐resilience section. Tianjun Ma wrote the section describing various cybersecurity threats. Emilie Soisson wrote the section on ethics. Joshua Yung worked on the figure in the network configurations section. Bruce Curran added a paragraph in the responsibilities section and provided comments and edits throughout the rest of the document.

## CONFLICT OF INTEREST STATEMENT

The Chair of the Working Group on Cybersecurity has reviewed the required Conflict of Interest statement on file for each member of the Working Group on Cybersecurity and determined that disclosure of potential Conflicts of Interest is an adequate management plan.

The members of the Working Group on Cybersecurity listed below attest that they have no potential Conflicts of Interest related to the subject matter or materials presented in this document: Ramon Alfredo Siochi, Peter Balter, Samantha Hedrick, Tianjun Ma, Emilie Soisson, Joshua Yung, and Bruce Curran.

The members of the Working Group on Cybersecurity listed below disclose the following potential Conflict(s) of Interest related to subject matter or materials presented in this document: Jonathan Howe, Varian Employee (AOS).

## APPENDIX A HIT RESPONSIBILITY CHECKLIST

### A.1

HIT is responsible for working with other stakeholders to recommend and implement cyber resilience strategies that address system failures and deliberate or accidental actions. HIT must recognize that ideal cyber security measures may conflict with ideal patient care and should have flexibility and secondary security measures in place to allow deviations from the ideal. HIT should also ensure that patient care systems, or approved alternatives used during cyber events, continue to perform properly. Table A1 describes a set of baseline responsibilities and the BCP phase where they are important:

**TABLE A1:** HIT responsibilities for cybersecurity.

Category / Phase	Responsibility
User authorization /Prevention	Manage systems that validate credentials and assign appropriate access rights and privileges.^40^
	Use two‐factor authentication (2FA) if the medical device supports it.
	Limit access to the needs of the user.
	Update access lists when users leave their role.
Server management / Prevention	Regularly patch and test server operating systems as allowed by the vendor (some patches may not be compatible with the vendor's device), verify functionality with the department that uses the server, and rollback the operating system patch if needed.
	Restrict network traffic to and from the server to known systems on specific ports.
	Limit operations to those essential for business.
	Use anti‐virus software and encryption that is compatible with the vendor's device. Consult with the medical physicist and vendor.
Server management / Preparation	Develop redundant servers and failover processes.
Server management/ Response	Isolate affected servers. Switch to backup servers.
Server management / Recovery	Reconstruct, decontaminate, and repair affected servers. Transfer data from backup servers to primary servers. Bring primary servers online. Assess the extent of data loss.
Storage management / Prevention	Design the storage architecture so that no single point of failure can cause a loss of connectivity or data.
Storage management / Preparation	Backup stored data. Deploy file level recovery systems to restore data to its state at a known good time point.
	Collaborate with the medical physicist to determine the number and frequency of “snap shots” for the backup process.
	Maintain off‐site and off‐line encrypted backups.
	Manage USBs / CDs/ DVDs so that they don't introduce malware on systems where they are used. Work with medical physicists to develop safe data transfer protocols with these removable storage systems where medical devices require them for the transfer of treatment plans and images.
Storage management / All phases	Data stored on cloud platforms requires close coordination with the cloud service provider.
Storage management / Response	Switch over to the backup storage. Assess the extent of data loss. Inform the department of the switch over and potential data loss. Assist the clinic in moving treatment plans to linear accelerators for offline mode treatments.
Storage management / Recovery	See server management/Recovery.
Network / Prevention	Place clinical equipment on isolated sub‐networks limiting connectivity to the general facility wide area network (WAN).
	Restrict connection of unknown devices using network access control (NAC) or other means.
	Regularly patch network infrastructure including switches, routers, and firewalls for known vulnerabilities.
Network / Preparation	Develop and deploy an efficient process to register new devices in a timely fashion to enable machine repairs and upgrades that require changing equipment.
	Eliminate single points of failure in the network infrastructure, possibly by using redundant systems.
	Discuss the use of DNS and DHCP on restricted networks with medical physicists and vendors of medical devices attached to the network.
Network / Response	Coordinate with medical physicists and the vendors of medical devices on the network to ensure efficient provision of connectivity on backup and failover systems.
Network / Recovery	Restore connectivity of medical devices on the network.
Database / Prevention	Establish security and user access rules.
Database / Preparation	Determine means and schedules for backups and snap shots, and for restoring the database to a second location.
Database / Response	Switch to backup databases.
Database / Recovery	Reconcile backup and primary databases to keep as much clean data as possible. Cleanup primary databases and the servers that host them.
Application support / All phases	HIT should ensure there are application support specialists focused on software covered in this document (i.e., TPS, OIS, Image review systems, etc.). These specialists should have vendor training where applicable for back‐end configuration and support and should work with medical physics and other clinical staff to configure and maintain the applications to ensure safety and optimize efficiency. In some cases, these personnel may be part of the MPs team or department staff, with joint responsibilities shared with HIT. They should receive training from the vendors of the applications on how to configure the applications for use during a cyber‐attack.
Security / Prevention	Perform security reviews of new and existing software and a systems vulnerability analysis. In‐house developers should work with Hospital IT to make sure that the appropriate development environment and processes are set up to reduce the risk of introducing vulnerabilities that leave one open to cyberattacks.

## APPENDIX B GENERAL FRAMEWORK AND USE CASE EXAMPLE

### B.1

The NIST Cybersecurity Framework^22^ is a very comprehensive and detailed document, which requires great effort to implement in its entirety; a faster way to improve one's cybersecurity risk profile is to focus on the four phases of a BCP and put them in the context of cybersecurity. Table B1 shows the phases and corresponding steps. Table B2 analyzes the UVM incident based on the general BCP framework in Table B1, by mapping the actions in Table B1 to the events in the incident. As an exercise, the reader could perform an analysis on the other incidents in Section 3, or on any cyber‐attack they may have experienced in their own department.

**TABLE B1:** Business continuity planning phases and general actions.

Phase	Action
A. Prevention	Educate and train staff on policies and procedures that reduce the risk of a cyberattack. Deploy systems to detect and remove threats. Design networks and systems to be robust to cyber‐attacks.
B. Preparation	Create data flow diagrams with all departmental devices. Include the software applications on those devices, and the IP addresses and ports on which they communicate. Inspect the data flow diagrams for vulnerabilities to cyber‐attacks and develop mitigations. Create diagrams of clinical workflows as they are currently practiced. Include the software applications used at each step of the workflow. Develop a downtime communications plan for staff and patients. Develop the failover plan to switch operations to the failover systems (alternative devices and networks on backup systems). Using the diagrams of data flows and clinical workflows, develop alternative offline and failover workflows (i.e., clinical workflows that use the failover systems). Use Ethics considerations when designing the alternative workflows. Proactively triage patients.
C. Response	Isolate the threat. Deploy the failover plan. Determine the affected clinical workflows and identify the corresponding alternative offline and/or failover workflows, and triage patients to the various alternatives. Communicate the response plan to all staff, including estimated time frames for each of the steps. (i.e., specify when to use offline workflows, when to use failover workflows, and when to return to normal workflows). Deploy the response plan.
D. Recovery	Restore all systems to normal operations. Reconcile patient charts. Modify or develop policies, procedures, and infrastructure to address the vulnerabilities that made it possible for the cyberattack to happen.

**TABLE B2:** Analysis of the UVM cyberattack. Applicable BCP framework sections from Table B1 are placed in brackets in the analysis column.

Phase	Analysis
Prevention	A work laptop was used to access personal email. Policies about mixing personal and work systems and accounts need to be designed or enforced. Employees should be educated and periodically reminded about these policies, or education with updates needs to be done when there is an increased threat level, and cyber threats need to be monitored more frequently. Updates should be applied to work laptops before the employees take them home. [A.1] Additionally, HIT should develop policies and procedures to (1) ensure all work laptops are updated and include the latest versions of anti‐virus protection, and (2) perform real‐time scanning of embedded objects in email attachments and other objects, encrypted or otherwise, that could be downloaded, saved, or otherwise find their way into the network. [A.2] A Unix‐based TPS was unaffected, as the malware was windows based. [A.3] The CT scanner could still acquire images. [A.3]
Preparation	There was no updated hard copy contact list for patients [B.4] DICOM files of CT scans were rerouted direct to the TPS. [B.1, B.2, B.5, B.6] Descriptions of the event do not provide a failover infrastructure. [B.5, B.6] Patients were not proactively triaged. [B.7]
Response	Hospital IT immediately shutdown the internet and the EMR when they started receiving user complaints and realized they were under attack. [C.1] The department was told to prepare for an extended downtime. [C.4] Patients were triaged to another site, treated with offline operation of the linac, or delayed. [C.3] System of checklists and safety rules were developed for offline treatments. [B.6, C.3] Standalone ROIS was set up, with treatment plans from the TPS. Prior treatment information was determined from patient conversations and calendars. [C.3] Descriptions of the event do not discuss a backup ROIS system. [B.5, C.2, C.5]
Recovery	Computers and servers were brought back online as they were cleared during cyber‐forensics investigations. Radiology viewing, the legacy ROIS database and the EMR were restored. [D.1] There was no mention of reconciling the standalone ROIS that was setup during the downtime with the legacy ROIS database. [D.2]
